# Serum interleukin-6 level as an early marker of injury severity in trauma patients in an urban low-income setting: a cross-sectional study

**DOI:** 10.1186/s12873-015-0048-z

**Published:** 2015-09-16

**Authors:** Paul K. Okeny, Peter Ongom, Olivia Kituuka

**Affiliations:** Department of Surgery, Gulu Regional Referral and Teaching Hospital, P.O Box 160, Gulu, Uganda; Department of Surgery, College of Health Sciences, Makerere University, Mulago Hill road, P.O Box 7072, Kampala, Uganda

**Keywords:** Interleukin-6, Kampala Trauma Score II, Duration of injury

## Abstract

**Background:**

Trauma is still the leading cause of death in many regions of the world. Severity scores have been developed to assist in management of trauma victims. Immune response to trauma has been known to positively correspond to the severity of trauma. Part of this response involves release of cytokines into blood circulation which promote the acute inflammatory response commonly seen after trauma. Studies have shown that IL-6 levels commonly correlate positively with the Injury Severity Score (ISS). The aim of this cross-sectional study was to determine whether this kind of relationship exists between IL-6 levels and injury severity in trauma patients in Mulago Hospital as defined by the Kampala Trauma Score (KTSII) which is locally developed.

**Methods:**

Trauma patients aged ≥18 years presenting to the Accident and Emergency unit of Mulago National Referral Hospital (MNRH) within 12 h after injury were recruited into the study after obtaining consent. Severity of injury was determined as per the Kampala Trauma Score (KTSII) and venous blood drawn for assay of serum IL-6 levels. Data obtained was entered and analyzed using Stata version 11 software focusing on the association between Serum IL-6 levels with Severity of trauma and duration of injury.

**Results:**

A total of 159 patients were recruited (79 Mild and 80 Severe trauma) with a male to female ratio of 4.7:1. Road traffic crashes (67.92 %) were the commonest cause of injury. Serum IL-6 levels were found to positively correspond with severity of injury (z = 4.718, p < 0.001). There was no significant correlation between serum IL-6 levels and duration of injury in both severe (r = 0.12, p = 0.29) and mild (r = 0.06, p = 0.62) trauma groups of patients. Only 9.43 % of trauma patients were brought in an Ambulance.

**Conclusions:**

Serum IL-6 levels correspond with severity of injury. However, within the first twelve hours after injury, these levels did not vary significantly with duration of injury.

## Background

Trauma remains the leading cause of death globally hence its public health significance. It affects mainly the young and most economically productive age groups [1–3]. This burden and death rates are higher in Africa than Europe due to factors like poor road designs and poor mechanical condition of motor vehicles [4]. In Uganda, trauma is the single most common reason for admission to surgical wards [5–7], affecting mainly the young population [8, 9].

Many severity scores have been developed to help in triage, management and prediction of outcome in trauma patients [10–12]. These scores are based on anatomical or physiological parameters or both. Immune response to injury as determined by circulating levels of serum IL-6 have been found to correspond to severity of injury as defined by the Injury Severity Score [13–16].

Damage associated molecular patterns (DAMPs) and alarmins trigger the immune response to trauma and/or burns in humans [17]. Xiao et al. [18] described this physiologic response as a ‘genomic storm’ characterized by increased gene expression and alteration of up to 80 % of the leukocyte transcriptome. The magnitude of the storm was found to be directly proportional to the frequency and severity of complications. It is now known that both pro and anti-inflammatory responses occur simultaneously as previously suggested by Xiao et al. [18] and are associated with injury severity and outcome [19].

These findings support the current paradigm shift towards use of biochemical/immunological markers in injury severity scoring [20] since many previous scores arguably depend on subjective anatomical and physiological parameters. Interleukin 6 is preferred because of its long duration of detectability in blood, independence of kidney function and availability of rapid measurement systems [21].

The aim of this study was to determine the association between serum IL-6 levels and injury severity as defined by the Kampala Trauma Score (KTS) II, a locally developed severity score tool in an Urban, low-income setting and describe the association between these levels and duration of injury.

## Methods

This was a cross-sectional study carried out at Mulago National Referral Hospital (MNRH) over a period of 2 months from January to February 2014.

### Study setting

The study was carried out at the Accident and Emergency (A&E) unit of MNRH which is headed by a consultant surgeon. This department handles both medical and surgical emergencies in separate wings. It has two operating rooms, an X-ray and Ultrasound facility, two resuscitation rooms, two mechanical ventilators and a twenty six bed holding emergency ward. Next to the department are a blood bank, haematology, microbiology and chemistry laboratories.

### Study participants

All trauma patients aged ≥18 years presenting or referred within 12 h of injury to MNRH were eligible for the study. Patients who from history or assessment were found to be on steroid therapy, asthmatic, or have diabetes mellitus or rheumatoid arthritis were excluded because these conditions affect serum IL-6 levels. Using the KTS II, patients were divided into two groups: severe injury (KTS II ≤8) and mild injury (KTS II >8).

Consecutive sampling was used for the severely injured group as opposed to systematic random sampling for the mildly injured group. The A&E department receives twenty two mildly injured patients per day (range 15 – 30). Study participants were recruited four days per week and with a study period of 2 months; every ninth mildly injured patient was consented and recruited into the study.

### Study procedure

On arrival patients were assessed based on the Advanced Trauma Life Support (ATLS) principles and scored using the KTS II. Consenting patients had variables age, sex, mode of arrival, duration of injury (time in hours from injury to time blood sample was drawn for analysis of serum IL-6 levels), type of injury, cause of injury and body region injured recorded.

Under aseptic technique, 4mls of venous blood was drawn from a convenient peripheral vein, centrifuged at 1000 r.p.m and the serum layer was removed and stored at -80 °C at the Makerere University College of Health Sciences Immunology Laboratory. Serum assay for IL-6 levels were run at the end of the study period using Human IL-6 ELISA kit supplied by Biolegend [22]. Repeated freeze-thaw cycles were avoided as recommended by the manufacturer.

### The Kampala Trauma Score

The most widely used injury scores are the ISS, Trauma Injury Severity Score (TRISS) and the RTS. These international scores are however very demanding and require detailed data collection and an analysis of accurate prospectively collected data. In 1996 the Injury Control Centre-Uganda (ICC-U) developed the Kampala Trauma Score I (KTSI) as a simplified version and composite of both the ISS and RTS so that it could be used by all health cadres in resource limited centers. In 2004, the score was revised from the initial 16-point scale to the current 10-point scale which is simpler to use. This revised version is now referred to as the KTSII. The KTSII considers and scores five parameters in patients’ assessment: age (in years), respiratory rate, systolic blood pressure, neurologic status and score for serious injuries on admission [Table 1]. Mild, moderate and severe injuries are designated scores 9 – 10, 7 – 8 and ≤6 respectively.

**Table 1:** The Kampala Trauma Score II

	Description	Score
A	Age (in years)	
	5-55	1
	<5 or >55	0
B	Systolic Blood Pressure on admission	
	More than 89 mm Hg	2
	Between 89-50 mm Hg	1
	Equal or below 49 mm Hg	0
C	Respiratory rate on admission	
	0-29/minute	2
	30+	1
	< or =9/minutes	0
D	Neurological status	
	Alert	3
	Responds to verbal stimuli	2
	Responds to painful stimuli	1
	Unresponsive	0
E	Score for serious injuries	
	None	2
	One injury	1
	More than one	0

Kampala Trauma Score total = A + B + C + D + E

The KTS has previously been validated and found to compare favorably with other trauma scores [7, 23–26].

### Ethical approval

Ethical approval was obtained from the department of surgery MNRH and the Institutional Review Board of Makerere University College of Health Sciences – School of Medicine. Written informed consent was sought from participants or their next of kin.

### Data analysis

Data were analyzed using Stata version 11. Descriptive analyses were presented using scatter plots, box plots and bar graphs. Summary statistics were presented using percentages for categorical variables, and median and interquartile ranges for continuous variables. Transformation methods were used for non-normally distributed data. The Mann-Whitney U test was used to assess for differences in IL-6 level between severe trauma and mild trauma. We log transformed both the IL-6 level and duration of injury to achieve a linear relationship. Pearson’s correlation coefficient *r* was used to assess the relationship of log transformed IL-6 and log transformed duration of injury. A one sided 2.5 % significance level was used for all analyses. Determination of sensitivity and specificity of serum IL-6 levels in stratifying patients by severity of trauma using the KTSII as gold standard was done. The cut off on serum IL-6 level was varied by 50 pg upwards starting from 1.00 pg and computed sensitivity and specificity at each level. Using the sensitivity and specificity scores, a receiver operator characteristic (ROC) curve was generated.

## Results

A total of 159 patients (79 mild injury and 80 severe injuries) were recruited into the study. Male to Female ratio was 4.7:1. Of the study participants, 42.14 % had studied up to primary school level and 11.32 % had no formal education [Table 2]. Only 9.43 % were brought in an Ambulance.

**Table 2:** Sociodemographic characteristics of study population by severity of trauma among trauma patients attending Mulago Hospital

Characteristic	Severe trauma	Mild trauma	*P*-value
	N = 80	N = 79	
Age, median(IQR)	27 (24-37)	27 (23-35)	0.70
Gender, n (%)			
Male	67 (83.75)	64 (81.01)	0.65
Education level, n (%)			
No formal Education	11 (13.75)	7 (8.86)	0.004
Primary	31 (38.75)	36 (45.57)	
Secondary	21 (26.25)	31 (39.24)	
Tertiary	5 (6.25)	5 (6.29)	
Occupation, n (%)			
Peasant	14 (17.50)	11 (13.92)	0.007
Business	28 (35.00)	33 (41.77)	
Salaried/wage earner	22 (27.50)	30 (37.97)	
Student	4 (5.00)	5 (6.33)	
Transport to Hospital, n (%)			
Ambulance	9 (11.25)	6 (7.59)	0.001
Police pickup	43 (53.75)	23 (29.11)	
Boda boda	3 (3.75)	18 (22.78)	
Ordinary(private/public) car	25 (31.25)	30 (37.97)	
Other	0 (0.00)	2 (2.53)	

The most commonly injured body region was head and neck [Table 3], accounting for 51.57 % of the study population. Road traffic crash was the commonest cause of injury accounting for 67.92 % and of these 45.37 % were pedestrians.

**Table 3:** Injury characteristics of study population by severity of trauma among trauma patients attending Mulago Hospital

Characteristic	Severe trauma, N = 80	Mild trauma, N = 79	*P*-value
Body region injured, n (%)			
Head and/or Neck	55 (68.75)	27 (34.18)	<0.001
Face	15 (18.75)	19 (24.05)	0.45
Thorax	17 (21.25)	11 (13.92)	0.23
Abdomen/visceral pelvis	4 (5.00)	5 (6.33)	0.72
Extremities/bony pelvis	27 (33.75)	28 (35.44)	0.82
External (Skin)	30 (37.50)	30 (37.97)	0.95
Cause of injury, n (%)			
Road traffic crush	57 (71.25)	51 (64.56)	0.14
Assault	19 (23.75)	15 (18.99)	
Falls	3 (3.75)	10 (12.66)	
Burns	1 (1.25)	3 (3.80)	
Type of injury, n (%)			
Penetrating	32 (40.00)	30 (37.97)	0.59
Blunt force	47 (58.75)	46 (58.23)	
Burn	1 (1.25)	3 (3.80)	
Role of crash victim, n (%)			
Driver	14 (17.50)	13 (16.46)	0.36
Passenger	13 (16.25)	20 (25.32)	
Pedestrian	29 (30.00)	20 (25.32)	

**Fig. 1:**
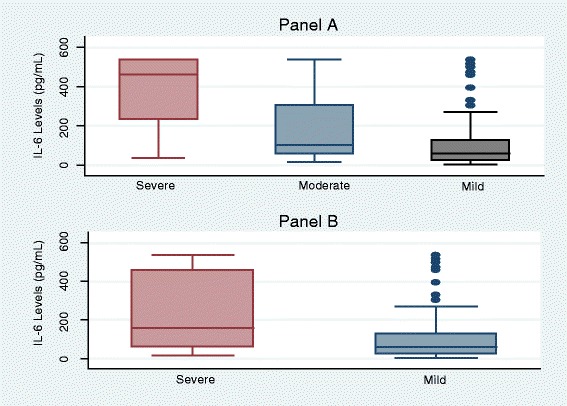
Distribution of IL-6 levels by severity of trauma among trauma patients attending Mulago Hospital. Panel **a**: Trauma categorized by KTS II into Severe vs Moderate vs Mild trauma. Panel **b**: Trauma categorized into Severe (combined KTS II severe and moderate trauma) vs Mild trauma

**Fig. 2:**
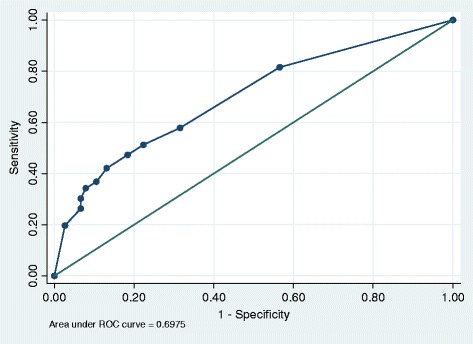
Receiver Operator Characteristic (ROC) curve for Serum IL-6 levels

There was a significant distribution of serum IL-6 levels among the severely injured and mildly injured groups of patients (z = 4.718, p < 0.001) [Fig. 1]. The area under receiver operator curve was 0.6975 (95 % CI 0.616 – 0.778) [Fig. 2]. At a cut off of 60 pg/ml, serum IL-6 levels were able to discriminate between mild and severe injury with a sensitivity of 77.63 % and specificity of 50.00 % with a Positive Predictive value of 60.82 and Negative Predictive value of 69.09.

**Fig. 3:**
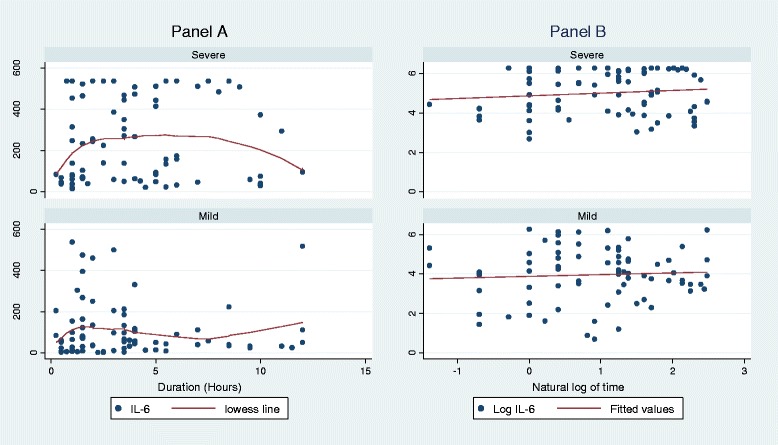
Scatter plots showing variation of IL-6 levels with duration of injury in hours in the Severe and Mild categories of trauma patients attending Mulago Hospital*.* Panel **a**: IL-6 levels vs duration of injury in hours. Panel **b**: Correlation between IL-6 levels and duration of injury in hours after Log transformation

The median duration of injury in severely injured patients was 3.5 (1.5-6.0) hours while that in the mildly injured group was 3.0 (1.25-4.5) hours. There was no significant correlation between serum IL-6 levels with duration of injury (severe group r = 0.12, p = 0.29 and mild group r = 0.06, p = 0.62) [Fig. 3].

## Discussion

The aim of this study was to describe the association between serum IL-6 levels and severity of injury as defined by the KTS II. Severe trauma included a KTS II score of ≤8 while Mild trauma included a KTS II score of >8.

We found a significant distribution of serum IL-6 levels among the severe and mildly injured groups of patients (z = 4.718, p < 0.001). Patients with severe trauma had a higher rank (Mann-Whitney test). Even when the KTS II was broken down to its three original categories of mild, moderate and severe trauma, serum IL-6 levels were still noted to increase with increasing/worsening severity of injury, showing that IL-6 levels in this study were relatively able to group trauma patients into severity categories.

Interleukin 6 is a 26 kDa cytokine produced as part of the human body’s immune response to trauma [27]. It is released with 1 – 4 h following trauma and stays for a few days before beginning to decline [28]. Its effects include but are not limited to B cell maturation, differentiation and activation, activation of Natural Killer cells and release of soluble Tumor Necrosis Factor receptor antagonist [27, 28]. These effects arise following interaction with IL-6 receptor system that is composed of a ligand binding α-subunit (IL-6R) and a signal transducing β-subunit (gp130) [27]. It therefore has both pro and anti-inflammatory effects much as it is grouped under Th1 cytokines [29].

Controlled and balanced inflammatory response is beneficial to the body. Excessive anti-inflammatory response is thought to cause immunoparalysis and hence worse outcomes. While measuring cytokines and adhesion molecules in the first 72 h in severely injured patients, Sousa et al. [19] found that serum IL-6 levels correlated with injury severity and outcomes. In their study, a low Th1/Th2 ratio was associated with worse outcomes.

Numerous authors elsewhere have documented that serum IL-6 levels positively correspond with severity of injury [14, 19, 30–34].

The area under the ROC curve was only 0.6975, showing that serum IL-6 levels were unable to significantly discriminate between severe and mild injury. However, at a cut off level of 60 pg/ml, serum IL-6 levels could differentiate between severe and mild injury with a relatively high sensitivity of 77.63 %. This is however higher than sensitivity of 16.7 % of IL-6 for MODS that was found by Frink et al. [35]. This difference could be because in this study, calculations were done at a serum IL-6 cut off level of 60 pg/ml so as not to miss any patient with severe injury.

Despite the fact that these studies used the ISS and where done in high-income countries with a modern health care system while we used the KTS II in a low-income setting/population with a prevalence of malnutrition at 19 % [36], serum IL-6, an inflammatory marker, was still found to positively correspond with injury severity. It may also reiterate the fact that the KTS II compares favorably with the ISS.

In this study, duration of injury of atleast 3 h is way beyond the recommended ‘golden hour’ in trauma management. Therefore, patients with severe life threatening injuries may have died before arrival at the emergency unit leading to a selection bias. Similar durations were reported in studies done in low-income settings [5, 8] compared to less than 70 min in high income settings [14, 37]. This could be due to a relatively poor Ambulance system in our setting since only 9.43 % of trauma patients arrived at the hospital in an Ambulance.

We did not find any significant correlation between serum IL-6 levels and duration of injury as compared to studies done elsewhere [14, 30]. This disparity could be that these studies measured serial serum IL-6 levels in each individual trauma patient as early as from the scene of injury whereas in this study, we analyzed IL-6 levels in different individuals who reported at different times to the hospital. Individuals may vary in the timing and magnitude of their physiobiochemical response to injury.

The median duration of injury in both trauma groups were within the six hours of peak levels found by Antunes et al. [32] and twelve hours of peak levels found by Gebhard et al. [30]. This means that the rise in serum IL-6 levels in this study population is probably only due to severity of trauma and not variation in time from injury to presentation.

### Study limitations

Due to the study design, we are unable to completely guarantee stability of serum IL-6 levels within the first few hours after injury. The median duration of injury (time to hospital), was 3 h. This brings in a selection bias since patients with life threatening injuries may have died within the first hour after injury.

## Conclusions

Serum IL-6 levels correspond with severity of injury as defined by the KTS II. Routine measurements of serum IL-6 levels in all trauma patients may be negated by costs in many centers. However, prognostic significance of serum IL-6 levels in severely injured patients in low-income settings needs to be investigated.

## Abbreviations

ISS: Injury Severity Score
RTS: Revised Trauma Score
TRISS: Trauma Injury Severity Score
KTSII: Kampala Trauma Score two
MNRH: Mulago National Referral Hospital
IL-6: Interleukin-6
DAMPs: Damage associated molecular patterns
ATLS: Advanced Trauma Life Support
A&E: Accident and Emergency
